# Spatial Hurdle Models for Predicting the Number of Children with Lead Poisoning

**DOI:** 10.3390/ijerph15091792

**Published:** 2018-08-21

**Authors:** Zhen Zhen, Liyang Shao, Lianjun Zhang

**Affiliations:** 1 School of Forestry, Northeast Forestry University, Harbin 150040, China; zhzhen@syr.edu; 2 Department of Forest and Natural Resources Management, State University of New York College of Environmental Science and Forestry, Syracuse, NY 13210, USA; shaoliyang@gmail.com

**Keywords:** overdispersion, zero-inflated count data, negative binomial Hurdle model, generalized linear mixed models, random effects, spatial effects

## Abstract

*Objective* The purpose of this study is to identify the high-risk areas of children’s lead poisoning in Syracuse, NY, USA, using spatial modeling techniques. The relationships between the number of children’s lead poisoning cases and three socio-economic and environmental factors (i.e., building year and town taxable value of houses, and soil lead concentration) were investigated. *Methods* Spatial generalized linear models (including Poisson, negative binomial, Poisson Hurdle, and negative binomial Hurdle models) were used to model the number of children’s lead poisoning cases using the three predictor variables at the census block level in the inner city of Syracuse. *Results* The building year and town taxable value were strongly and positively associated with the elevated risk for lead poisoning, while soil lead concentration showed a weak relationship with lead poisoning. The negative binomial Hurdle model with spatial random effects was the appropriate model for the disease count data across the city neighborhood. *Conclusions* The spatial negative binomial Hurdle model best fitted the number of children with lead poisoning and provided better predictions over other models. It could be used to deal with complex spatial data of children with lead poisoning, and may be generalized to other cities.

## 1. Introduction

Lead exposure has adverse health impacts on children’s mental and physical growth and development. Children under six years of age have the highest chance of exposure to lead hazard due to their behavior and other factors. Even low exposure will affect children’s nerve and brain system, causing low IQ, learning disabilities, and hearing problems [1,2]. Water, paint, soil, and dust are the common sources of environmental lead exposure. However, pediatric lead poisoning is completely preventable through an intervention such as proactively identifying the lead sources in the environment and educating parents and guardians on how to reduce exposure to these environmental sources [3]. The local health departments started lead screening programs in the early 1990s to monitor the children’s blood lead levels. Later, in order to prevent pediatric lead poisoning, public health service agencies decided that it is important to shift from case identification and management to primary prevention. Thus, these service agencies are interested in developing analytical methods to identify high-risk areas of lead poisoning.

Statistical models are useful tools for assessing the risks of lead poisoning. They establish the relationships between the number of lead poisoning cases and socio-economic variables and/or environmental factors. These models then can be applied to identify and predict localized risks. Griffith et al. [4] developed a spatially filtered logistic regression model to explain the empirical probabilities (i.e., the number of children with an elevated level of lead poisoning divided by the number of cases) from the blood lead surveillance records (1992 to 1994) [4]. They found that population density, household rent/house value, and the percentage of African American people were significant risk factors for children’s lead poisoning [4]. However, after 20 years of intervention implementation by the Onondaga county government, New York, the lead poisoning incidences have been reduced to approximately 4% and the disease has become rare. As a result, the outcome variable moved from a binomial frequency distribution to a Poisson frequency distribution [5], because Poisson can be used to approximate a binomial distribution if the population size is sufficiently large and the probability of the event is sufficiently small. Therefore, it would be more appropriate to use Poisson regression to model the number of children with lead poisoning cases.

In epidemiological research, most public health data involve response variables of disease counts, proportions or rates rather than continuous variables [6]. Generalized linear models (GLIM) provide a collection of modeling methodologies, extending from Gaussian distribution to a family of exponential distributions including the binomial and Poisson distributions [7,8]. GLIM consists of a random component defining the distribution of error terms, a systematic component defining the linear combination of explanatory variables, and a link function defining the relationship between the systematic and random components [6]. For the cases of rare diseases, Poisson models are appropriate for modeling count data [9,10].

Spatial count data are more challenging for statistical modeling. This type of data commonly presents the problems of overdispersion and spatial effects (spatial autocorrelation and heterogeneity) [11]. Overdispersion is concerned with the strict requirement of the Poisson distribution that the mean and variance of a count response variable be equal. For rare disease count data, the observed sample variance is usually much larger than the sample mean because zero counts tend to occur more often than a higher number of counts. This is particularly true for spatially clustered data such as the counts of events in a census tract due to spatial heterogeneity within and between small geographical areas. If the overdispersion in the count data is ignored, it results in underestimating the standard errors of regression coefficients and leading to biased hypothesis testing [7,8]. Spatial autocorrelation is concerned with the dependence of a response variable among adjacent neighboring units in a geographical area. Thus, one of the model assumptions, i.e., independent observations, is violated, which has a strong impact on the standard error estimation of regression coefficients. However, the impact is complicated depending on the sign and magnitude of spatial autocorrelation. Positive spatial autocorrelation causes the underestimation of the standard errors, while negative spatial autocorrelation leads to their overestimation [12].

Agresti [6] extended the generalized linear models (GLIM) to generalized linear mixed models (GLIMM) by including random effects, which can be used to handle overdispersion and clustered observations. Haining et al. [11] proposed two modeling strategies to deal with spatial autocorrelation and overdispersion such as the conditional spatial autoregressive model (spatial CAR model) and a spatial filter model, which provided a robust approach for statistical inferences. Ma et al. [13] applied a generalized linear mixed Poisson model to investigate the influence of the climate and land cover patterns of birds by taking spatial autocorrelation and heterogeneity into account.

Further, the overdispersion problem may become more difficult when the count data contain excessive zero counts. Such data are not uncommon in many public health applications, especially spatial count data due to the spatial partitioning of the study areas [11,14]. Given the restriction of equal mean and variance, a Poisson distribution may not be able to handle the count data with disproportionately too many zeros (a.k.a. zero-inflated count data). Other statistical models are more appropriate such as a negative binomial model, a Hurdle model, and a zero-inflated model [15,16]. The negative binomial model is a mixture of gamma distribution and Poisson model. It relaxes the assumption of equal mean and variance in a Poisson model by including a dispersion parameter to accommodate the unobserved heterogeneity in the count data [7,8]. Mullahy [17] proposed Hurdle models in order to model the count data with excessive zeros. It has two-parts: the first part is a logistic regression to model the probability that a count is zero or a positive integer value, and the second part is a truncated-at-zero distribution to model the number of counts greater than zero (i.e., positive integer numbers). Although Mullahy [17] limited his discussion to the case where both transition probabilities were from zero to one and the count variables were Poisson variates, the truncated distribution can be either a Poisson or a negative binomial. Later, Lambert [18] proposed zero-inflated models which are a mixture of a logistic regression and either a Poisson or a negative binomial model. The logistic model is used to separate zeros from positive counts and the Poisson or negative binomial model is used to model the positive counts. Zero-inflated models are distinct from Hurdle models in their way of interpreting and analyzing zero counts. Zero-inflated models assume that zero observations have two different origins, namely “structural” and “sampling”, while Hurdle models assume that all zeros are from one “sampling” source [7,8,19].

The objective of this study was to develop a model to predict the expected number of children with lead poisoning cases with an elevated blood lead level (≥10 µg/dL) in the inner city of Syracuse, New York, USA. The census block was chosen as the spatial resolution to identify the residential areas of children with lead poisoning. In our previous studies [20], we assumed that the children with lead poisoning risk could be assessed through two perspectives, physical exposure to the environmental sources of lead hazard and socio-economic vulnerability. We explored a number of socio-economic statuses and environmental factors [4,21] and identified three important factors (i.e., soil lead concentration, building year and the town taxable value of the residential houses) as the predictors used in the regression models in this study. Because the number of children with lead poisoning has been greatly reduced after 20 years of lead poisoning prevention, the available count data were strongly skewed with excessive zero counts. Hence, we attempted to compare different statistical models to obtain an optimal model for the given empirical dataset and assess the model prediction performance by the jackknife resampling technique.

## 2. Materials and Methods

### 2.1. Materials

In this study, the response variable was the number of children having blood lead levels (BLL) ≥ 10 µg/dL (a.k.a. elevated BLL). The Onondaga County Health Department (OCHD) provided the surveillance data of the children’s BLL. The dataset included the children’s lead screening tests from 2007 to 2011 in Syracuse, New York. To avoid the sampling bias caused by the follow-up blood tests, we chose only the first test of each child in the surveillance dataset. The lead test records included children’s birthday, test date, test type, gender, race, and primary address. There were a total of 24,222 observations in the data. All the records were geo-coded by the reference data 2010 TIGER/Line Shapefiles provided by US Census Bureau. We merged the observation database with the census block map layer in ArcGIS 10 (http://resources.arcgis.com/en/home/), and found out that a total of 1393 blocks have records of children screened for lead levels, out of the total 2350 blocks in the city of Syracuse, NY.

**Figure 1:**
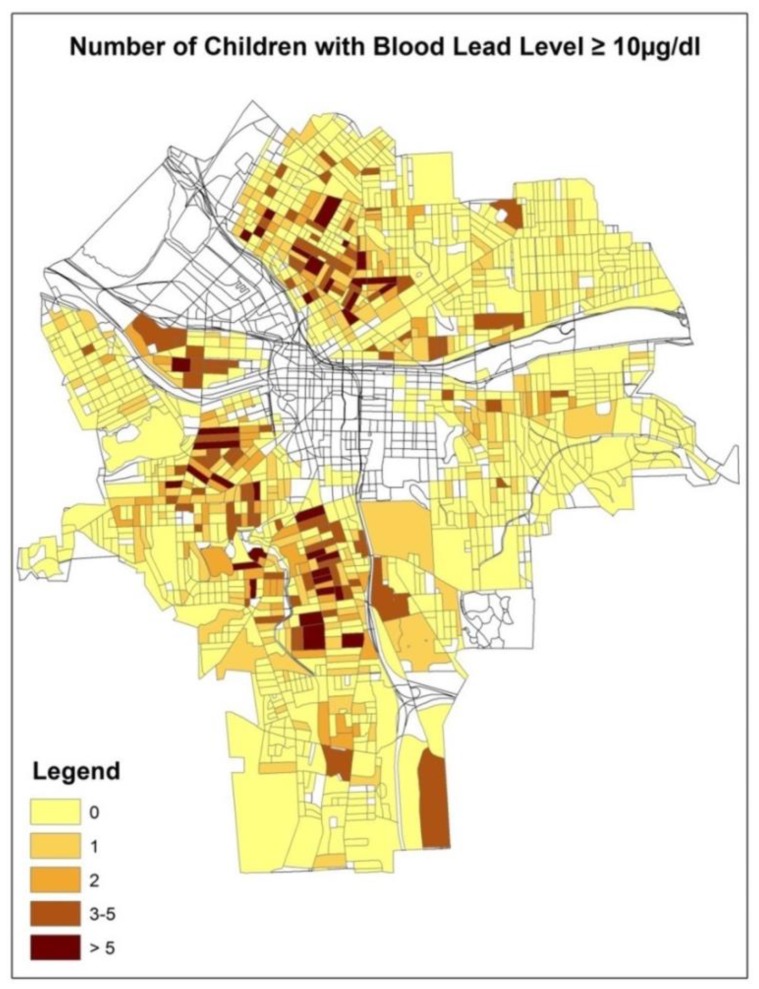
The spatial patterns of the number of children with a blood lead levels (BLL) ≥ 10 µg/dL at the census block level in the city of Syracuse, New York (NY).

**Figure 2:**
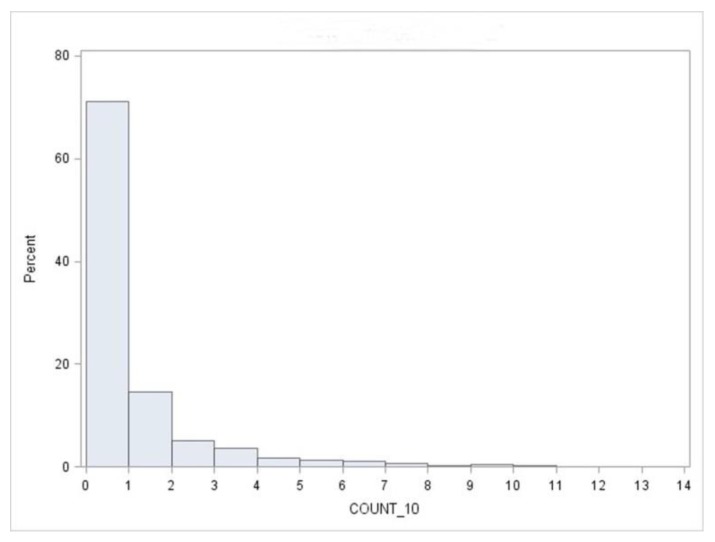
The frequency distribution of the number of children with a BLL ≥ 10 µg/dL (Count_10) at the census block level in the city of Syracuse, NY.

We counted the number of children whose BLL ≥ 10 µg/dL in each census block. If there was no child having the BLL ≥ 10 µg/dL, we recorded a 0 for the census block (Figure 1). Otherwise, the actual number of children with the BLL ≥ 10 µg/dL was recorded. The sample mean of the response variable was 0.68, while the sample variance was 2.32 (Table 1). Thus, the sample variance was more than three times greater than the sample mean. The frequency distribution of the children’s BLL ≥ 10 µg/dL showed excessive zeros and was highly skewed to the right, which also confirmed the underlying overdispersion (Figure 2).

Three predictor variables were used in this study based on the variable selection process in the previous study [20], including the building year of the houses (i.e., the year of construction), the town taxable value of the houses (dollar), and the soil lead contamination concentration (parts per million, ppm). The building year and town taxable value of the houses were collected from the Syracuse-Onondaga County Planning Agency 2005 tax parcel layer. We selected the residential properties in the city of Syracuse by the land-use code. The building years and town taxable values of the houses in a census block were then averaged to get the data in ArcGIS 10 (http://resources.arcgis.com/en/home/). The oldest residential buildings were built in 1860, and the newest was built in 1978 (Table 1). The town taxable value showed that the average taxable value was $58,445 and the median taxable value was $54,874. The lowest taxable value was $14,000 and the highest was $230,106 (Table 1). Then, we divided the town taxable value by 1000, and took a natural log-transformation of the results to generate the predictor variable.

Soil samples were collected and analyzed by Johnson and Bretsch in the summer of 2003 and 2004 [22]. There were a total of 3000 soil samples taken across the city of Syracuse. Distance inverse weighting interpolation was employed to obtain a continuous surface of the soil lead concentration. In addition, the average soil lead concentration was computed at the census block level using ArcGIS 10 (Environmental Systems Research Institute, Inc., Redlands, CA, USA). The soil lead concentration was highly skewed, the sample mean of the soil lead concentration was 186 ppm, while the sample median was 165 ppm (Table 1). We took a natural log transformation on the soil lead concentration as the predictor variable.

### 2.2. Methods

The response variable was the number of children having a BBL ≥ 10 µg/dL in each census block. However, many geographical units did not have any lead poisoning cases. The empirical data did show the problem of excessive zeros. To identify the “best” model for the count response variable in this study, we attempted to compare several potential candidate models, including a Poisson model (as the benchmark), a negative binomial model, and Hurdle models. The Hurdle models were used because we assumed the zero counts were only “sampling zeros”, i.e., the zero counts in the census blocks having records of children screened for lead levels. On the other hand, if the zero counts (i.e., structural zero) also included the children without BLL screening tests, the zero-inflated models would be a good choice, which treat the zero counts generated by two different processes [7,8]. In addition, we utilized the census blocks as the random effects to handle the spatial effects across the study area [11,15].

### 2.3. Poisson Model

Poisson regression is the traditional method for modeling count response variables (Y*_i_*), although it is usually criticized for its strict assumption of equal mean and variance. We used the Poisson regression as a benchmark model for our children’s BLL data. The Poisson distribution is defined as [7,8]:

(1) $$P\left(Y_{i}\right)=\frac{e^{-\mu_{i}}\cdot \mu_{i}^{Y_{i}}}{Y_{i}!}$$

where P(Y*_i_*) is the probability that the number of events (Y*_i_*) occurred in a census tract (*i* = 1, 2, …, *n*), and μ*_i_* is the parameter representing the expected value of Y*_i_*. It is assumed that mean E(Y*_i_*) = μ*_i_* and variance Var (Y*_i_*) = μ*_i_*. The set of predictor variables X influences μ*_i_* via the function:

(2) $$\ln\left(\mu_{i}\right)=X_{i}\beta$$

where β is the set of regression coefficients to be estimated from the data, and ln stands for the natural logarithm. In addition, the census blocks were used as the random effects to incorporate the spatial autocorrelation among the census blocks:

(3) $$\ln\left(\mu_{i}\right)=X_{i}\beta +Z_{i}\gamma$$

where Z*_i_* is the design matrix for the census blocks and γ is the random effects parameter such that γ ~ N (0, G) [7].

### 2.4. Negative Binomial Model

The negative binomial model is considered the most common alternative to the Poisson model [7,8], and addresses the overdispersion by including a dispersion parameter to accommodate the unobserved heterogeneity in the count data:

(4) $$P\left(Y_{i}\right)=\frac{\Gamma \left(Y_{i}+\frac{1}{\kappa }\right)}{\Gamma \left(Y_{i}+\frac{1}{\kappa }\right)\cdot \Gamma \left(\frac{1}{\kappa }\right)}{\left(\frac{1}{1+\kappa \mu }\right)}^{\frac{1}{\kappa }}{\left(\frac{\kappa \mu }{1+\kappa \mu }\right)}^{Y_{i}}$$

with the mean E(Y*_i_*) = μ and variance function Var (Y*_i_*) = μ + κμ^r^, where κ is known as the dispersion parameter. The Poisson model is the limiting model of the negative binomial mode when κ→0. The link function of the negative binomial model was also:

(5) $$\ln\left(\mu_{i}\right)=X_{i}\beta$$

Similar to the Poisson model, the census blocks were used as the random effects below:

(6) $$\ln\left(\mu_{i}\right)=X_{i}\beta +Z_{i}\gamma$$

### 2.5. Hurdle Models

The Hurdle model is a widely used alternative for the count data with excessive zeros and has a two-stage modeling process. In the first stage, a logistic regression is used to model a binary variable, which measures whether the count response variable is zero or a positive integer. In other words, the observed counts fall below or above the hurdle (i.e., zero). Then, a truncated-at-zero model is used to explain the observed counts greater than zero [7,8]. For the count response variable Y*_i_*, let’s define two independent random variables Y_1_ and Y_2_ such that:

(7) $$P\left(Y=y\right)=\{\begin{array}{c} P\left(Y_{1}=y\right)\;\mathrm{if}\;y=0\\ \left[1-P\left(Y_{1}=0\right)\right]\cdot \frac{P\left(Y_{2}=y\right)}{1-P\left(Y_{2}=0\right)}\;\mathrm{if}\;y\geq 1 \end{array}$$

Because Y_1_ is essentially used to establish the event of crossing the hurdle (i.e., zero), we defined P(Y*_i_* = 0) = p*_i_*, and P(Y*_i_* ≥ 1) = 1‒ p*_i_*, and used a logistic regression to model p*_i_*. For the positive integer counts, a truncated-at-zero probability density function can be used, such as a truncated Poisson or truncated negative binomial [7,8,17]. In this study both the truncated Poisson and negative binomial distributions were utilized. The link functions for the two-stages are:

For the logistic regression:

(8a) $$\ln\left(\frac{p_{i}}{1-p_{i}}\right)=X_{i}\alpha$$

For the truncated model:

(8b) $$\ln\left(\mu_{i}\right)=X_{i}\beta$$

where α and β are the sets of regression coefficients for the stages 1 and 2, respectively. Note that different predictor variables X could be used in each of the two-stage models because the predictor variables affected the first stage might not be the same as those affecting the second stage. However, we used the same three predictor variables for both two-stage models. Again, we extended the Hurdle model to spatially clustered count data by including the random effects parameters to account for the spatial autocorrelation among the census blocks:

For the logistic regression:

(9a) $$\ln\left(\frac{p_{i}}{1-p_{i}}\right)=X_{i}\alpha +{Z\gamma }_{1}$$

For the truncated model:

(9b) $$\ln\left(\mu_{i}\right)=X_{i}\beta +{Z\gamma }_{2}$$

where γ_1_ and γ_2_ were the random effects parameters for the stages 1 and 2, respectively.

### 2.6. Offset Modeling

For geographically distributed count data, the offset is important for modeling the count response variable because it often has “units”. For example, the number of children’s lead poisoning cases in the census block *i* might be larger than that in the census block *j* because the block *i* had a substantially larger children population than the block *j*. It is necessary to take different “population sizes” into account [11]. Therefore, the offset is an adjustment for the population sizes and accounts for heterogeneity due to differences in population size. In this study, we used the total number of BLL screening tests (N*_i_*) in each census block as the offset variable. The count response variable (i.e., the number of children with a BLL ≥ 10 µg/dL) was divided by the offset variable as the adjustment so that the response variable in the modeling process was actually the rate of children having a BLL ≥10 µg/dL in each census block. It could be used to compare different census blocks with different population sizes (i.e., the total number of BLL screening tests). Consequently, the link functions above modeled the rates of children’s lead poisoning (e.g., Equation (2)) became:

(10) $$\ln\left(\frac{\mu_{i}}{N_{i}}\right)=\ln\left(\mu_{i}\right)-\ln\left(N_{i}\right)=X_{i}\beta$$

(11) $$\ln\left(\mu_{i}\right)=X_{i}\beta +\ln\left(N_{i}\right)=X_{i}\beta +{\mathrm{offset}}_{i}$$

The regression coefficient for the offset was fixed at 1 [11].

### 2.7. Model Specification

The three predictor variables are denoted as follows: (1) X_1_ = building year of houses, (2) X_2_ = natural logarithm of town taxable value (in thousands of dollars), and (3) X_3_ = natural logarithm of soil lead concentration (ppm). Therefore, the link function for the Poisson and negative binomial models (as well as the truncated model of the Hurdle models) is:

(12) $$\ln\left(\mu_{i}\right)=X_{i}\beta +\ln\left(N_{i}\right)=\beta_{0}+\beta_{1}\cdot X_{1}+\beta_{2}\cdot X_{2}+\beta_{3}\cdot X_{3}+\ln\left(N_{i}\right)$$

where $\beta =\left[\beta_{0},\beta_{1},\beta_{2},\beta_{3}\right]$ is the set of regression coefficients to be estimated from the data. Similarly, the link function for the logistic regression of the Hurdle models is:

(13) $$\ln\left(\frac{p_{i}}{1-p_{i}}\right)=X_{i}\alpha =\alpha_{0}+\alpha_{1}\cdot X_{1}+\alpha_{2}\cdot X_{2}+\alpha_{3}\cdot X_{3}$$

where $\alpha =\left[\alpha_{0},\alpha_{1},\alpha_{2},\alpha_{3}\right]$ is the set of regression coefficients to be estimated from the data.

### 2.8. Model Assessment

The Akaike information criterion (AIC) was used to evaluate the model fitting in this study [23]. AIC is commonly formulated as:

(14) AIC=−2logL+2p

where logL is the maximum log-likelihood representing the goodness of model fitting and p is the number of model parameters to be estimated [23]. The model with a smaller AIC is preferred. If the difference of the AIC values between two models is >2, the difference between the two models is considered significant (some people may use other criteria/cut-off such as >4, >7, >10, or >14) [23]. After the model fitting, the model predictions were produced and summarized into count categories. The predicted frequency distributions from the four models were compared with the observed frequency distribution of the response variable to compare and illustrate the model fitting.

To assess the model performance, the four models were validated by jackknife resampling [24], in which each model was constructed using all but one observation (sample size n – 1). Afterward, the fitted model was used to predict the value of the count response variable for the excluded observation. Two statistics based on jackknifing were obtained for each model to assess the model prediction performance (Equations (15) and (16)) for each model as follows:

Mean prediction error (MPE):

(15) $$\mathrm{MPE}=\frac{\sum_{i=1}^{n}\left(Y_{i}-{\hat{Y}}_{i,-i}\right)}{n}$$

Mean absolute error (MAE):

(16) $$\mathrm{MAE}=\frac{\sum_{i=1}^{n}\left|Y_{i}-{\hat{Y}}_{i,-i}\right|}{n}$$

where $Y_{i}$ is the *i*th observed count response variable, and ${\hat{Y}}_{i,-i}$ is the predicted value of the *i*th observed value by the fitted model that was fitted using (n − 1) observations and that excluded the use of the *i*th observation. Then, the model jackknifing predictions (${\hat{Y}}_{i,-i}$) were summarized into count categories, and Pearson χ^2^ was used to compare the observed frequencies of $Y_{i}$ with the jackknifing predictions.

In this study, the PROC NLMIXED procedure in SAS 9.4 (SAS Institute, Inc., Carey, NC, USA) was used for fitting all models and for conducting jackknifing model validation [25].

## 3. Results

In general, the soil lead concentration was negatively associated with the building year with a Pearson correlation coefficient of −0.54, meaning that the older the house, the higher the lead concentration in the soil. The Pearson correlation between the house building year and its town taxable value was 0.35, indicating that older houses had lower taxable values. The Pearson correlation coefficient between the town taxable value and soil lead concentration was −0.33. Given the relatively lower correlations between the three predictor variables, there was no multicollinearity problem in the modeling and prediction processes [26].

The Poisson, negative binomial, Poisson Hurdle, and negative binomial Hurdle models were fitted to the children’s BLL data, respectively, with the census blocks as the random effects (RE) and the total number of BLL screening tests (N*_i_*) in each census block as the offset variable (Equation (12)). The above four generalized linear mixed models with the random effects (i.e., the GLIMM models) fitted the data significantly better than the corresponding generalized linear models without the random effects (i.e., the GLIM models). For example, the AIC of the Poisson model without RE was 3009.7, while the AIC of the Poisson model with RE reduced to 2810.5. The AIC of the negative binomial model with RE was 2586.9, which was smaller than that (2637.9) of the negative binomial model without RE. In general, the magnitudes of the model coefficients of the GLIMM models were smaller than those of the GLIM models, but the standard errors of the model coefficients in the GLIMM models became slightly larger than those of the GLIM models, though all model coefficients remained statistically significant at the significance level of *α* = 0.05.

In this study, we used the census blocks as the random effects of the four regression models to deal with the spatial effects [11]. We computed Moran’s I, one of the measures on spatial autocorrelation for the response variable [12], as well as the model residuals in order to demonstrate the improvement of model fitting if the random effects (RE) were incorporated into the models. The results indicated that for the observed count response variable, the Moran’s I was 0.0529 (Z = 37.78 and *p* < 0.0001), for the residuals of the Poisson model without RE the Moran’s I was 0.0204 (Z = 14.86 and *p* < 0.0001), and for the residuals of the Poisson model with RE the Moran’s I was reduced to 0.00261 (Z = 1.33 and *p* = 0.1832). The results clearly indicated that using the census blocks as RE would be able to significantly reduce the spatial autocorrelation in the model residuals.

The negative binomial model with RE fitted the children’s BLL data better than the Poisson model with RE (Table 2). The estimated model coefficients were in a close range between the Poisson model and the negative binomial model, but the standard errors (SE) of the negative binomial model coefficients were much larger than those of the Poisson model (Table 3). The dispersion parameter of the negative binomial model was estimated as κ = 1.1424, which was significantly different from zero. Thus, the negative binomial model allowed the variance of the response variable to be 2.1424 (= 1 + 1.1424, Equation (4), assuming linear variance function) times larger than the mean of the response variable so that it relaxed the rigorous assumption of equal mean and variance for the Poison model. The estimated coefficients of the negative binomial model were all statistically significant. The model coefficient for X_1_ (building year) indicated that more recently built houses would have a lower likelihood of lead poisoning. The coefficient of X_2_ (log of town taxable value) showed that the higher the average block town taxable value, the lower the chance of having lead poisoning. The estimated parameter of X_3_ (log of soil lead concentration) implied that the children would have a higher chance to exposure to lead if the surrounding of the house had higher soil lead concentration (Table 3).

The Hurdle models had two parts: (1) a logistic regression was used to predict the probability that the count response variable was zero or a positive integer, and (2) a truncated (Poisson or negative binomial) model was used to model the observed counts greater than zero (i.e., positive integers). The negative binomial Hurdle model fitted the children’s BLL data better than the Poisson Hurdle model (Table 2). The model coefficients in both the Poisson and negative binomial Hurdle models were very similar for both logistic regression and truncated models (Table 4). However, all model coefficients were statistically significant in the logistic regression models, while the model coefficients for the X_3_ (log of soil lead concentration) in both truncated models became non-significant (*p*-value > 0.11), indicating that the soil lead concentration was less important for predicting the positive cases of the children’s BLL.

**Figure 3:**
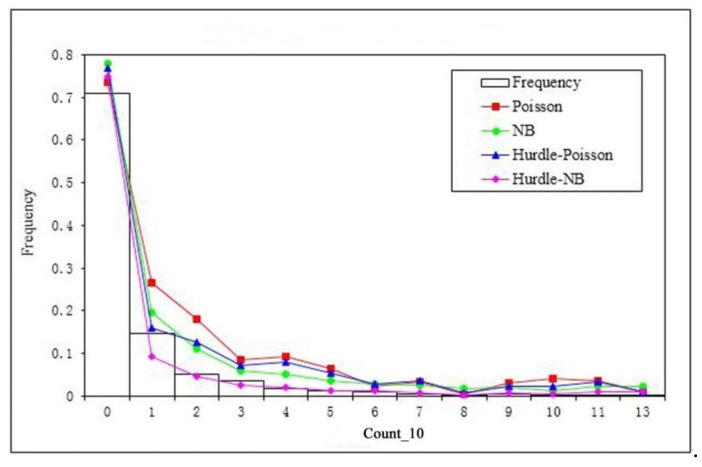
The comparison of model fitting from the four models against the observed frequency of children’s BLL ≥ 10 µg/dL (Count_10).

After the model fitting, the four models were used to produce the predictions of the expected numbers of children’s BLL ≥ 10 µg/dL at the census block level, and the Spearman correlation coefficients were computed between observed and predicted response variable. The results indicated that the Spearman correlation coefficient for the Poisson model was 0.53, negative binomial model 0.53, Poisson Hurdle model 0.44, and negative binomial Hurdle model 0.46. Then, the predicted numbers of children’s BLL ≥ 10 µg/dL at the census block level were aggregated to the frequencies of children’s BLL ≥ 10 µg/dL across the range of the count response variable (0 to 13 in this study). Figure 3 shows the comparison between the observed and predicted frequencies of the response variable. It was evident that the Poisson, negative binomial, and Poisson Hurdle over-predicted the frequency at each count category, while the negative binomial Hurdle model provided the best model fitting (Figure 3). The Pearson χ^2^ statistic was calculated between observed and predicted frequencies for each model. The results showed that the Poisson model with RE had the largest deviance from the observed frequencies (χ^2^ = 65.45), followed by the Poisson Hurdle model with RE (χ^2^ = 26.50) and the negative binomial model with RE (χ^2^ = 21.02). The negative binomial Hurdle model with RE (χ^2^ = 6.62) had the smallest deviance from the observed frequencies. The results indicated that the negative binomial Hurdle model handled both issues of overdispersion and excessive zeros effectively and improved model fitting.

**Figure 4:**
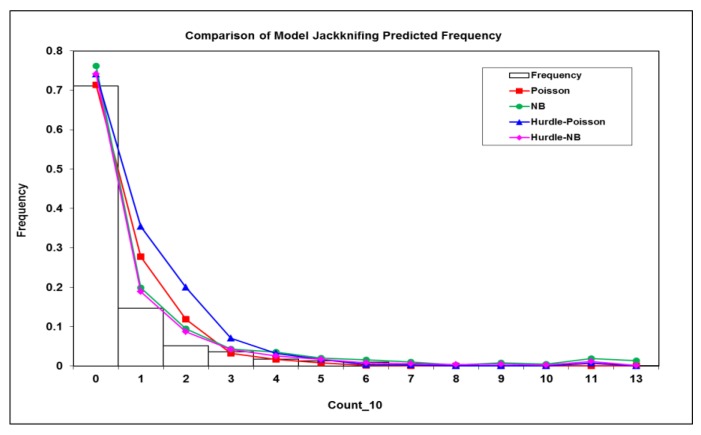
The comparison of model jackknifing predictions from the four models against the observed frequency of children’s BLL ≥ 10 µg/dL (Count_10).

The jackknife resampling was used to validate the model performance or prediction for the four models. The mean prediction error (MPE) and mean absolute error (MAE) were calculated (Table 5). It appeared that two negative binomial models produced a smaller MPE and MAE than two Poisson counterparts, indicating that negative binomial models were more suitable to deal with overdispersion better. Further, the model jackknifing predictions were summarized into count categories and compared with the observed frequency of the response variable (Figure 4). It was evident that both the negative binomial and negative binomial Hurdle models performed much better than both Poisson and Poisson Hurdle models, which over-predicted the frequency at the count categories of 1, 2, and 3 (Figure 4). The Pearson χ^2^ statistic was then calculated between jackknifing predicted and the frequencies for each model were observed. The results showed that the negative binomial Hurdle model with RE had the smallest χ^2^ deviance from the observed frequencies (Table 5), which confirmed that the negative binomial Hurdle model handled both issues of overdispersion and excessive zeros effectively and improved both model fitting and prediction.

## 4. Discussion

Our results showed that the negative binomial Hurdle model with the random effects was the best model among the four GLIMM models applied in this study. Generally, the Poisson model was not a good candidate model for the count data with overdispersion due to its restricted assumption of equal mean and variance. In this case, the negative binomial model became a better alternative because it included a dispersion parameter to deal with the overdispersion. The Hurdle model, a mixture of logistic regression and truncated-at-zero model, is designed to treat the zero count and positive counts in two stages when there are excessive zero counts in the data. It was no surprise to find that the negative binomial Hurdle model was the best model for our children’s BLL data because it could account for the overdispersion from the excessive zeros. Other research results showed that the zero-inflated negative binomial model had a similar performance as the negative binomial Hurdle model, and both showed better model fitting and prediction over other candidate models for the vaccine adverse health outcomes modeling [16]. In this study, however, the excessive zeros only came from one process (i.e., sampling zeros), which was the children’s BLL less than 10 µg/dL. Thus, the Hurdle model would be more appropriate to handle the excessive zeros. On the other hand, if the zero counts also included the children without BLL screening test, the zero-inflated negative binomial would be a good choice, which treats the zero counts generated by two different processes [7,8,16]. It could be used to deal with complex spatial data of children with lead poisoning and may be generalized to other cities or conditions.

It was argued that the lead in soil was a major lead contamination source causing pediatric lead poisoning. Therefore, soil lead was considered as one of the covariates in this study. Among the four models, the soil lead concentration was statistically significant in the Poisson and negative binomial models. It was also significant in the logistic regression models of the two Hurdle models for predicting the probability of children having lead poisoning in a given census block. However, it became less significant in the truncated models for predicting the number of children at lead poisoning risk. Therefore, the soil lead concentration may play a different role in children’s lead poisoning. It may be more important for identifying the possibility of children’s lead poisoning, but it may not be strongly associated with high risks of lead poisoning. This result might indicate that relatively poor conditions of older houses with lower tax values may be a more important factors impacting the high risks of children’s lead poisoning, while the soil lead contamination may have a certain level of association with indoor dust but this pathway was influenced by latent factors, so we concluded that soil lead had an insubstantial link with the high risks of children with lead poisoning [1,2,3].

Lead-contaminated dust and lead-based paint appeared to be the principal sources of exposure for young children. Water could be contaminated by the leaching of lead from lead pipes or lead-based solder at pipe joints. Lead abatement treatments and water pipe systems were expensive for a low-income household to implement and replace. Given these lead pathways, the children living in older, inner-city housing and low socioeconomic class are at higher risks for lead poisoning. Our models clearly showed that the building year of the houses is an important risk factor of children with lead poisoning because old houses are associated with lead-based paint as well as lead pipes or pipe joints in the water delivery system. Another important risk factor is the town taxable value of the houses which implies the ability to mitigate the lead hazard. Low-income households will not have been able to remodel and are more vulnerable to the lead hazard.

One of the limitations of this study was that the data sources were from different years, for example, the response variable was collected in 2007–2011, the socio-economic variables such as the building year and town taxable value were collected from the 2005 tax layer, and the soil lead concentration data were collected in 2003–2004. The timing mismatch of the data and variables may cause inaccuracies and imprecisions in the modeling process.

## 5. Conclusions

This study demonstrated that the spatial negative binomial Hurdle model fitted the children’s BLL count data best and provided better predictions over other models. The spatial effects (spatial autocorrelation and heterogeneity) were modeled using the random effects across the census blocks, which provided a better estimation of the standard errors of the model coefficients. The application of the offset variable enabled to take into account the different “population sizes” (the total number of BLL screening tests in each census block) so that the models provided the rate of lead poisoning for children at risk. The building year and town taxable value of the houses were statistically significant for predicting the lead poisoning at multiple risk levels, while the soil lead concentration was less significantly associated with high risks of children’s lead poisoning. This finding may provide the insight that the effective lead poisoning prevention intervention and mitigation strategy might focus on the tap water lead testing and continued lead-based paint inspection, especially in the older houses with relatively poor conditions.

## Figures and Tables

**Table 1:** The descriptive statistics of the variables used in this study.

Variable	Mean	Median	Variance	Minimum	Maximum
Number of Children with BLL ≥ 10 µg/dL	0.68	0	2.32	0	13
Total number of BLL tests	31.05	28.00	430.03	1	207
Building Year of Houses	1923	1922	308.6	1860	1978
Town Taxable Value (dollar)	58,445	54,874	567,148,162	14,000	230,106
Soil Lead Concentration (ppm)	185.8	164.5	12,660	10.03	840.8

Note: BLL stands for blood lead level.

**Table 2:** The summary of the −2 log likelihood and AIC of the four regression models.

Model	−2 Log Likelihood	AIC	Number of Parameters
Poisson with RE	2800.5	2810.5	5
Negative Binomial with RE	2574.9	2586.9	6
Poisson Hurdle with RE	2760.0	2780.0	10
NB Hurdle with RE	2671.9	2693.9	11

Note: RE stands for the random effects; AIC stands for the Akaike information criterion.

**Table 3:** The comparison of model fitting for the Poisson and negative binomial (NB) models.

Variable	Model Parameter	Poisson with RE		NB with RE	
		Estimate	SE	Estimate	SE
	${\hat{\beta }}_{0}$	31.814 ***	6.926	40.983 ***	9.853
Building Year	${\hat{\beta }}_{1}$	−0.018 ***	0.004	−0.022 ***	0.005
Town Taxable Value	${\hat{\beta }}_{2}$	−0.565 *	0.226	−1.051 ***	0.294
Soil Lead	${\hat{\beta }}_{3}$	0.317 **	0.092	0.404 **	0.134
	${\hat{\sigma }}^{2}$	0.861 ***	0.119	0.698 ***	0.119
	κ			1.142 ***	0.145

Note: RE stands for the random effects; SE stands for the standard error of model coefficient; The symbols *, **, and *** indicate statistical significance at *α* = 0.05, 0.01, and 0.001 levels, respectively.

**Table 4:** The comparison of model fitting for the Poisson Hurdle and negative binomial (NB) Hurdle models.

Variable	Model Parameter	Poisson Hurdle with RE		NB Hurdle with RE	
		Estimate	SE	Estimate	SE
	${\hat{\alpha }}_{0}$	31.273 **	11.143	25.016 *	11.099
Building Year	${\hat{\alpha }}_{1}$	−0.017 **	0.006	−0.014 *	0.006
Town Taxable Value	${\hat{\alpha }}_{2}$	−0.731 *	0.305	−0.787 *	0.304
Soil Lead	${\hat{\alpha }}_{3}$	0.588 ***	0.162	0.621 ***	0.162
	${\hat{\beta }}_{0}$	23.746 *	9.150	24.003	13.959
Building Year	${\hat{\beta }}_{1}$	−0.013 **	0.005	−0.013	0.007
Town Taxable Value	${\hat{\beta }}_{2}$	−0.562 *	0.247	−0.927 **	0.335
Soil Lead	${\hat{\beta }}_{3}$	0.182	0.112	0.288	0.179
	${\hat{\sigma }}_{1}^{2}$	0.693 ***	0.120	0.687 ***	0.119
	${\hat{\sigma }}_{2}^{2}$	0.439 ***	0.100	0.346 *	0.147
	κ			1.259 *	0.484

Note: ${\hat{\alpha }}_{0}$ ~ ${\hat{\alpha }}_{3}$ are the model coefficients for the logistic regression in the Hurdle models; ${\hat{\beta }}_{0}$ ~ ${\hat{\beta }}_{3}$ are the model coefficients for the truncated Poisson or NB Hurdle models; The symbols *, **, and *** indicate statistical significance at α = 0.05, 0.01, and 0.001 levels, respectively.

**Table 5:** The summary of jackknifing model validation for the four regression models.

Model	MPE	MAE	χ^2^
Poisson with RE	0.2107	0.7587	121.78
Negative Binomial with RE	0.1585	0.7436	8.52
Poisson Hurdle with RE	−0.5377	1.2119	49.26
NB Hurdle with RE	−0.3615	1.1095	4.11

Note: RE stands for the random effects.
